# Exome-wide association study identifies genetic polymorphisms of *C12orf51*, *MYL2*, and *ALDH2* associated with blood lead levels in the general Korean population

**DOI:** 10.1186/s12940-017-0220-x

**Published:** 2017-02-17

**Authors:** Sang-Yong Eom, Myung Sil Hwang, Ji-Ae Lim, Byung-Sun Choi, Ho-Jang Kwon, Jung-Duck Park, Yong-Dae Kim, Heon Kim

**Affiliations:** 1 Department of Preventive Medicine, College of Medicine, Chungbuk National University, 1 Chungdae-Ro, Seowon-Gu, Cheongju, Chungbuk 28644 Korea; 2 Food Risk Analysis Division, National Institute of Food and Drug Safety Evaluation, 187 Osongsaengmyeong 2-Ro, Heungdeok-Gu, Cheongju 28159 Korea; 3 Department of Preventive Medicine, Dankook University College of Medicine, 119 Dandae-Ro, Dongnam-Gu, Cheonan, Chungnam 31116 Korea; 4 Department of Preventive Medicine, Chung-Ang University College of Medicine, 84 Heukseok-Ro, Dongjak-Gu, Seoul, 06974 Korea

**Keywords:** Single nucleotide polymorphism, Blood lead level, Exome-wide association study, Aldehyde dehydrogenase 2

## Abstract

**Background:**

Lead (Pb) is a ubiquitous toxic metal present in the environment that poses adverse health effects to humans. Inter-individual variation in blood Pb levels is affected by various factors, including genetic makeup. However, limited data are available on the association between genetic variation and blood Pb levels. The purpose of this study was to identify the genetic markers associated with blood Pb levels in the Korean population.

**Methods:**

The study subjects consisted of 1,483 healthy adults with no history of occupational exposure to Pb. We measured blood Pb levels and calculated probable daily intake of Pb according to dietary data collected using 24-hour recall. We conducted exome-wide association screening using Illumina Human Exome-12v1.2 platform (*n* = 500) and a replication analysis using VeraCode Goldengate assay (*n* = 1,483).

**Results:**

Among the 244,770 single nucleotide polymorphisms (SNPs) tested, 12 SNPs associated with blood Pb level were identified, with suggestive significance level (*P* < 1 × 10^−4^). In the Goldengate assay for replication, three SNPs (*C12orf51* rs11066280, *MYL2* rs12229654, and *ALDH2* rs671) were associated with statistically suggestively significant differences in blood Pb levels. When stratified by drinking status, a potential association of *C12orf51* rs11066280, *MYL2* rs12229654, and *ALDH2* rs671 with blood Pb level was observed only in drinkers. A marginally significant gene-environment interaction between *ALDH2* rs671 and alcohol consumption was observed in relation to blood Pb levels. The effects of the three suggestively significant SNPs on blood Pb levels was dependent on daily calcium intake amounts.

**Conclusions:**

This exome-wide association study indicated that *C12orf51* rs11066280, *MYL2* rs12229654, and *ALDH2* rs671 polymorphisms are linked to blood Pb levels in the Korean population. Our results suggest that these three SNPs are involved in the determination of Pb levels in Koreans via the regulation of alcohol drinking behavior, and that their negative effects may be compensated by appropriate calcium intake.

**Electronic supplementary material:**

The online version of this article (doi:10.1186/s12940-017-0220-x) contains supplementary material, which is available to authorized users.

## Background

Lead (Pb) is a global environmental health hazard that poses substantial risk to humans. Epidemiologic evidence indicates that increased Pb exposure is associated with hypertension [1], peripheral vascular disease [2], increased adult mortality [3], reproductive impairment [4], renal impairment [5], and altered immune function [6]. Pb, a widely distributed metal, is a contaminant of ambient and workplace air, as well as of water, various foods, and tobacco [7, 8]. Diet and air are major sources of Pb exposure for the general population [9]. Absorption of Pb occurs mainly via the respiratory and gastrointestinal tracts [7, 9]. The rate of absorption of Pb via the gastrointestinal tract (10–15%) is relatively lower than via inhalation (up to 50%) [9]. Most Pb (about 90%) absorbed by the body accumulates in the skeleton and is slowly released from the bones [7, 9]. The individual nutritional statuses of iron, calcium, and zinc are known to be important factors that affect the gastrointestinal absorption of Pb [10, 11].

Inter-individual variation in blood Pb level is affected by various lifestyle and behavioral factors (i.e., smoking, drinking, dietary intake, and physical activity), as well as exposure dose [7, 9, 12–14]. In addition, genetic differences related to Pb toxicokinetics and toxicodynamics influence the body burden and toxic effects of this metal [13, 15]. Several genes, such as aminolevulinate dehydratase (*ALAD*) and vitamin D receptor (*VDR*) [16–18], are involved directly or indirectly in Pb toxicokinetics; however, there are relatively few studies assessing the effects of genetic factors on blood Pb levels. Recently, the first genome-wide association study (GWAS) for blood Pb levels, which was conducted in cohorts from Australia and the UK, confirmed that genetic variation for the *ALAD* gene plays a significant role in determining blood Pb levels [17]. However, to our knowledge, there is no study to identify a genetic marker for blood Pb levels in the Asian population, particularly in Korean individuals, using a genome-wide approach.

Therefore, the purpose of this study was to identify the genetic markers associated with blood Pb levels in the Korean population by exome-wide association screening and replication analysis.

## Methods

### Study subjects

The study subjects consisted of 1,483 healthy adults with no previous history of occupational exposure to Pb. The subjects were selected from a cohort established by the Korean Research Project on the Integrated Exposure Assessment of Hazardous Substances for Food Safety (KRIEFS). The characteristics of this KRIEFS cohort and the method used to select the study subjects are described in detail in previous studies [19, 20]. Trained interviewers obtained demographic information as well as data on lifestyle factors such as smoking history, alcohol drinking habits, and food consumption, through a structured questionnaire. Venous blood was collected from subjects for genotypic analysis and evaluation of blood Pb levels. The collected blood was stored at −80 °C, as aliquots, until experimental use. This study was approved by the Institutional Review Board of Dankook University Hospital, Republic of Korea (IRB No. 2013-03-008), and informed consent was obtained from all participants.

### Estimation of Pb intake

The probable daily intake of Pb was estimated from the dietary data collected using 24-hour recall. To determine the Pb content of the 135 food items, which represented the most frequently consumed food items based on Korean Health and Nutrition Examination Survey data, more than ten samples of each food item were gathered from seven metropolitan cities in South Korea. After pretreatment, the Pb content was measured using an inductively coupled plasma-mass spectrometer (ICP-MS, Perkin-Elmer, Elan 6100 DRC). The estimated amount of Pb intake for each food item was calculated by multiplying the food intake amount by its median content of Pb, and the estimated daily total intake of Pb was calculated by adding the Pb intake amounts for all food items.

### Analysis of blood Pb level

Blood Pb level was determined using a polarized Zeeman atomic absorption spectrophotometer (Model Z-2700, Hitachi, Tokyo, Japan). Briefly, blood was added to nitric acid and diluted with diammonium hydrogen phosphate and 1% Triton X-100, followed by vigorous mixing. The detection limit was 0.059 μg/dL for blood Pb. For samples with concentrations of blood Pb below the limit of detection, the concentration was substituted with the value for the limit of detection divided by the square root of 2.

### Genotyping analysis

#### Exome-wide association screening

Exome-wide association screening using an exome chip was performed to select SNPs associated with blood Pb levels in the Korean population. After randomly selecting 500 people from among the study subjects, exome-wide association screening was conducted using a Human Exome chip v1.2 (Illumina, San Diego, USA), in which 244,770 SNP markers may be analyzed simultaneously. Human Exome chip is a commercial genotyping chip containing about 220,000 nonsynonymous SNPs that have putative functional exonic variants selected from whole-genome sequences. It also contains some SNPs located in the promoter region and splice site and also including disease-related tag markers such as various cancers, type 2 diabetes, and metabolic diseases recently identified in GWAS.

From quality control of human exome chip data, 783 SNPs were not found to be in Hardy-Weinberg equilibrium (HWE) (*P* < 0.001), 309 SNPs had call rates less than 95%, and 211,808 SNPs had extremely low minor allele frequencies of less than 1% (including monomorphic). The average call rate of all the samples was greater than 99.9%, with a minimum value of 99.4%. A blind replication test was conducted on 20 randomly selected samples; the error rate of all the samples was less than 0.05%, and the average concordance rate was 99.96%. For the 32,743 SNPs located on autosomal chromosomes with MAF of more than 1%, sufficient call rates (>95%), and in HWE (*P* > 0.001), the association with the markers of blood Pb levels was evaluated by multiple regression analysis using the program PLINK, and 12 suggestively significant SNPs (*P* < 1 × 10^−4^) were selected. Linkage disequilibrium (LD) block was determined using Haploview, and Tag SNPs were selected from the identified haplotype block. SNPs for which a probe could not be designed for the Goldengate assay were excluded from the final replication analysis. Finally, four SNPs (rs1268474, rs11066280, rs12229654, and rs671) on chromosomes 1 and 12 were selected by exome-wide association screening.

#### Replication genotyping analysis

Replication genotyping analysis was performed for the four selected SNPs using the VeraCode Goldengate assay (Illumina, San Diego, CA, USA) in all subjects. Analysis was performed on all 1,483 samples that passed DNA quality control (QC). The average call rate of the samples was 99.4%, and that of the SNPs was 99.3%. Two samples with call rates of less than 95% were excluded from the final analysis (*n* = 1,481). The results of the blind replication test on 19 randomly selected samples were highly reproducible, with an average concordance rate of 99.5%.

### Statistical analysis

The concentration of blood Pb was log-transformed for statistical analyses as this parameter was not normally distributed. Means of blood Pb levels for various genotypes were compared by analysis of variance. To test the effects of SNPs on blood Pb levels, univariate and multivariate regression models, with covariates such as age, sex, smoking status, drinking habits, and dietary Pb intake, were used. The multivariate regression model analysis was performed with two covariate combinations. One included age, gender, and smoking status as covariates, and the other additionally included drinking status and dietary intake of Pb as variables in the first model. The correlation between dietary Pb intake and blood Pb levels was evaluated using the Spearman correlation coefficient. In addition, a stratification analysis was performed on drinking and calcium intake status to assess the effect of SNP on blood Pb level according to these two variables. The estimated amount of calcium intake was categorized into tertiles, and a stratified analysis was used to estimate the potential joint effects. *P*-values for interactions between the genotypes and dietary Pb or calcium intake were assessed, using the Wald test, for the cross-product term in a model containing the main effects of genotype and exposure variables. We used the Bonferroni correction for multiple tests (*n* = 32,743 tests) and set the statistical significance and suggestive threshold to *P*-values less than 1 × 10^−6^ and 1 × 10^−4^, respectively. Genetic association analyses were performed using PLINK v 1.07 software. A Manhattan plot of the exome-wide association study results was generated using Haploview 4.2 software. All other statistical analyses were performed using SPSS 23 (IBM, Armonk, NY, USA).

## Results

The dietary Pb intake and blood Pb level were 20.43 μg/day and 2.21 μg/dL, respectively, and significantly higher in males than in females. The levels of both parameters were highest in the group aged 50–59; blood Pb levels were found to increase significantly with age. The dietary Pb intake and blood Pb levels were higher in smokers than in non-smokers, and in drinkers than in non-drinkers (Table 1).

**Table 1:** General characteristics of the study subjects were included in final analysis

		*N* (%)	Dietary Pb (μg/day)	Blood Pb^a^ (μg/dL)
Total subjects		1,481	20.43 ± 9.31	2.21 (2.17–2.26)
Gender	Males	640 (43.2)	21.89 ± 10.10	2.60 (2.52–2.67)
	Females	841 (56.8)	19.31 ± 8.51	1.96 (1.91–2.01)
			*P* < 0.001	*P* < 0.001
Age groups	−29	254 (17.2)	18.34 ± 9.03	1.66 (1.59–1.74)
	30–39	265 (17.9)	19.80 ± 8.29	2.05 (1.97–2.14)
	40–49	341 (23.0)	21.63 ± 8.86	2.15 (2.07–2.24)
	50–59	331 (22.3)	21.77 ± 9.99	2.56 (2.47–2.66)
	60+	290 (19.6)	19.88 ± 9.76	2.66 (2.56–2.77)
			*P* < 0.001	*P* < 0.001
Smoking history	Non-smokers	962 (65.0)	19.99 ± 8.93	2.03 (1.98–2.08)
	Ex-smokers	243 (16.4)	21.90 ± 9.54	2.60 (2.48–2.72)
	Current-smokers	276 (18.6)	20.67 ± 10.28	2.61 (2.50–2.72)
			*P* = 0.015	*P* < 0.001
Alcohol use	Non-drinkers	361 (24.4)	19.36 ± 8.63	2.07 (1.99–2.14)
	Drinkers	1120 (75.6)	20.77 ± 9.50	2.26 (2.21–2.32)
			*P* = 0.009	*P* < 0.001

^a^Data presented as geometric mean with 95% confidence intervals

**Fig. 1:**
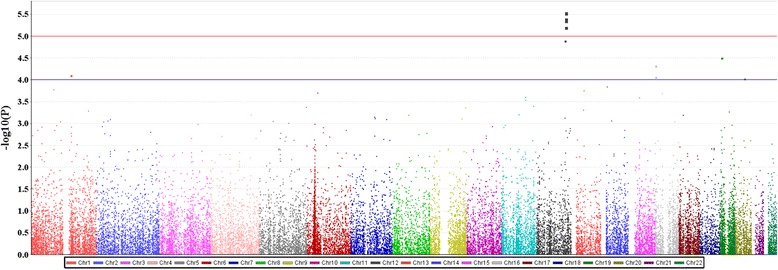
Manhattan plot of *P* values for exome-wide SNP association with blood lead levels; the *P* values in –log10 scale are plotted against the chromosomal locations of the SNPs. The *blue horizontal line* indicates the suggestive association level (*P* = 1 × 10^−4^). The *red horizontal line* indicates *P*-values of 1 × 10^−5^

To identify SNP markers associated with blood Pb levels, we performed an exome-wide association study of 244,770 SNPs via human exome chip analysis. The Manhattan plot, which was derived from the association analysis between blood Pb levels and SNPs using linear regression analysis with an additive genetic model, is shown in Fig. 1. The list of top 100 most significant SNPs associated with blood Pb level, as identified by exome-wide association screening, is provided in Additional file 1: Table S1. No SNP reached an exome-wide significance level of association (*P* < 1 × 10^−6^). SNPs associated with blood Pb levels, with suggestively significant level (*P <* 1 × 10^−4^), are listed in Table 2. Variant chromosome 12 open reading frame 51 (*C12orf51*) rs11066280 showed the strongest association (beta = −0.331, *P =* 2.88 × 10^−6^ in additive model). Out of these 12 SNPs, 4 SNPs were finally selected for the replication study using the Goldengate assay, through Tag SNP selection. The regional association plot of SNPs near *C12orf51* and aldehyde dehydrogenase 2 (*ALDH2*) genes on chromosome 12q24 is shown in the Additional file 1: Figure S1.

**Table 2:** The 12 single nucleotide polymorphisms associated with blood lead levels at a suggestive significance level (*P* < 1 × 10^−4^), as identified by exome-wide screening

rs ID	Chr.	Position	Nearest gene	SNP type	Variant allele	MAF	β^a^	SE	*P*-value
rs11066280	12	111302166	*C12orf51*	intronic	A	0.170	−0.331	0.070	2.88 × 10^−6^
rs2074356	12	111129784	*C12orf51*	intronic	A	0.140	−0.348	0.074	2.98 × 10^−6^
rs11066015	12	110652392	*ACAD10*	intronic	A	0.156	−0.329	0.071	3.97 × 10^−6^
rs671	12	110726149	*ALDH2*	nonsynonymous	A	0.155	−0.328	0.071	4.40 × 10^−6^
rs3782886	12	110594872	*BRAP*	synonymous	G	0.165	−0.319	0.070	6.27 × 10^−6^
rs12229654	12	109898844	*MYL2*	intergenic	C	0.139	−0.331	0.075	1.28 × 10^−5^
rs2228539	19	6877378	*EMR1*	nonsynonymous/splicing	G	0.015	0.903	0.215	3.18 × 10^−5^
rs2745099	16	1477459	*PTX4*	nonsynonymous	A	0.095	−0.354	0.086	4.80 × 10^−5^
rs41268474	1	150959136	*C1orf68*	nonsynonymous	A	0.058	0.441	0.111	7.90 × 10^−5^
rs2667672	16	1476381	*PTX4*	nonsynonymous	A	0.118	−0.317	0.080	8.76 × 10^−5^
rs2745097	16	1476500	*PTX4*	nonsynonymous	A	0.118	−0.317	0.080	8.76 × 10^−5^
rs6126559	20	35990690	*VSTM2L*	intronic	A	0.401	0.217	0.055	9.50 × 10^−5^

*Chr* chromosome, *MAF* minor allele frequency, *SE* standard error

^a^beta coefficient was adjusted for age, sex and smoking status by multiple linear regression analysis with an additive genetic model

Table 3 shows dietary Pb intake and blood Pb levels according to the genotypes for the four SNPs analyzed via the replication study. Dietary Pb intake levels were not found to vary by SNP. However, we observed significant differences in blood Pb levels according to the three SNPs [*C12orf51* rs11066280, myosin light chain 2 (*MYL2*) rs12229654, and *ALDH2* rs671]. Moreover, the Spearman correlation coefficients between dietary Pb intake and blood Pb levels differed across the genotypes for these three SNPs.

**Table 3:** Dietary lead intake, blood lead levels, and their correlation coefficient according to the four selected SNPs analyzed via the replication study

SNP ID	Gene symbol	Genotype	*N*	Dietary Pb	Blood Pb	Spearman’s correlation coefficients
rs41268474	*C1orf68*	GG	1284	20.34 ± 9.21	2.21 (1.48)	0.097^**^
		GA	193	20.51 ± 9.33	2.22 (1.49)	0.091
		AA	4	28.21 ± 15.93	2.12 (1.59)	0.712
				*P* = 0.960	*P* = 0.958	
rs11066280	*C12orf51*	TT	1001	20.36 ± 9.31	2.25 (1.49)	0.103^**^
		TA	433	20.38 ± 8.98	2.14 (1.46)	0.112^*^
		AA	47	20.80 ± 10.49	2.04 (1.42)	−0.121
				*P* = 0.951	*P* = 0.027	
rs12229654	*MYL2*	TT	1087	20.39 ± 9.33	2.25 (1.49)	0.127
		TG	353	20.31 ± 8.86	2.13 (1.46)	0.019
		GG	27	19.30 ± 10.38	1.92 (1.37)	−0.220
				*P* = 0.873	*P* = 0.015	
rs671	*ALDH2*	GG	1053	20.40 ± 9.33	2.26 (1.49)	0.120^**^
		GA	388	20.24 ± 8.86	2.11 (1.46)	0.064
		AA	40	21.32 ± 10.79	1.98 (1.38)	−0.210
				*P* = 0.773	*P* = 0.003	

*P<0.05, **P<0.01

After adjustment for potential confounders (e.g. age, sex and smoking status), three SNPs, namely *C12orf51* rs11066280, *MYL2* rs12229654, and *ALDH2* rs671, associated with blood Pb level with suggestive significance. In the case of *C12orf51* rs11066280, *MYL2* rs12229654, and *ALDH2* rs671 polymorphisms, blood Pb levels were found to decrease significantly as the number of variant alleles increased (Table 4).

**Table 4:** Statistical significance of replicated SNPs for blood lead levels according to the univariate- or multivariate- regression models from replication analysis

SNP ID	Gene symbol	Univariate			Multivariate model 1^a^			Multivariate model 2^b^		
		β	SE	*P*-value	β	SE	*P*-value	β	SE	*P*-value
rs41268474	*C1orf68*	0.011	0.072	0.878	0.085	0.062	0.168	0.090	0.061	0.144
rs11066280	*C12orf51*	−0.134	0.046	0.004	−0.170	0.040	1.94 × 10^−5^	−0.116	0.041	0.004
rs12229654	*MYL2*	−0.158	0.052	0.002	−0.184	0.044	3.46 × 10^−5^	−0.130	0.045	0.004
rs671	*ALDH2*	−0.172	0.048	3.74 × 10^−4^	−0.197	0.041	1.73 × 10^−6^	−0.139	0.043	0.001

SNPs were coded using an additive genetic model

^a^Adjusted for age, sex and smoking status

^b^Adjusted for age, sex, smoking status, alcohol drinking and dietary lead intake

The *C12orf51, MYL2,* and *ALDH2* SNPs were found to be highly related to alcohol consumption [21]; therefore, we performed stratified analyses to assess the association between the SNPs and blood Pb levels according to alcohol drinking status. In non-drinkers, the chromosome 1 open reading frame 68 (*C1orf68*) rs41268474 was associated with blood Pb level. However, *C12orf51* rs11066280, *MYL2* rs12229654, and *ALDH2* rs671 polymorphisms were associated with blood Pb level only in drinkers. Although not statistically significant, a weak interaction was observed between alcohol drinking and the *ALDH2 rs671* genotype with respect to blood Pb levels (*P*_for interaction_ = 0.067) (Table 5).

**Table 5:** Association between the replicated SNPs and blood lead levels according to alcohol consumption status

SNP ID	Gene symbol	Non-drinkers (*N* = 361)			Drinkers (*N* = 1120)			
		β^a^	SE	*P*-value	β^a^	SE	*P*-value	*P*_for interaction_
rs41268474	*C1orf68*	0.278	0.111	0.012	0.024	0.072	0.741	0.099
rs11066280	*C12orf51*	−0.068	0.062	0.275	−0.137	0.053	0.010	0.191
rs12229654	*MYL2*	−0.073	0.068	0.284	−0.157	0.058	0.007	0.369
rs671	*ALDH2*	−0.069	0.062	0.266	−0.182	0.057	0.002	0.067

SNPs were coded using an additive genetic model

^a^Adjusted for age, sex, smoking status and dietary lead intake

We additionally performed stratified analyses according to the dietary calcium intake, as calcium was related to both blood Pb level and alcohol drinking [22, 23]. Interestingly, in the high calcium-intake group, *C12orf51* rs11066280, *MYL2* rs12229654, and *ALDH2* rs671 polymorphisms were not associated with blood Pb levels. However, potential associations between these SNPs and blood Pb level were observed in the low calcium-intake group (Table 6).

**Table 6:** Association between the replicated SNPs and blood lead levels according to dietary calcium intake

SNP ID	Gene symbol	Dietary calcium intake, mg/day									*P*_for interaction_
		Low (<473) (*N* = 494)			Medium (473–760) (*N* = 493)			High (>760) (*N* = 494)			
		β^a^	SE	*P*-value	β^a^	SE	*P*-value	β^a^	SE	*P*-value	
rs41268474	*C1orf68*	0.046	0.111	0.679	0.131	0.098	0.181	0.078	0.111	0.484	0.776
rs11066280	*C12orf51*	−0.147	0.073	0.043	−0.126	0.072	0.081	−0.066	0.069	0.335	0.661
rs12229654	*MYL2*	−0.154	0.077	0.047	−0.141	0.082	0.084	−0.089	0.078	0.253	0.821
rs671	*ALDH2*	−0.165	0.077	0.033	−0.155	0.074	0.037	−0.094	0.072	0.190	0.797

SNPs were coded using an additive genetic model

^a^Adjusted for age, sex, smoking status, drink and dietary lead intake

## Discussion

In the present study, we identified three SNPs (i.e., *C12orf51* rs11066280, *MYL2* rs12229654, and *ALDH2* rs671) on chromosome 12 that were associated with blood Pb levels in the Korean population. These three SNPs have been previously shown to be associated with alcohol consumption [21, 24], and are involved in the determination of Pb body burden in the Korean population via regulation of alcohol drinking behavior, especially in individuals with low calcium intake. To the best of our knowledge, this study presents the first evidence of a potential association between genetic factors and Pb body burden in the Asian population, using exome-wide association screening.

To date, several studies have performed genome-wide analyses to study the effects of genetic factors on individual differences in blood Pb concentration [17, 25, 26]. A previous genetic linkage analysis for 2,962 Australian adult twins reported that genetic variation with respect to the near solute carrier family 4, sodium bicarbonate cotransporter, member 7 (*SLC4A7*) gene locus on chromosome 3 plays an important role in determining blood Pb concentration, after excluding shared environmental effects [25]. The first GWAS for cohorts from the UK and Australia identified the *ALAD* rs1805313 polymorphism on chromosome 9 affects blood Pb levels [17]. However, other GWAS have reported that no SNPs reached the threshold for statistical significance (*P* < 4.5 × 10^−9^) [26]. The finding of an association between *SLC4A7* and blood Pb level was not replicated by other studies. On the other hand, some studies have reported that *ALAD* genetic polymorphisms are associated with Pb body burden or toxicity, as the enzyme encoded by this gene, which is involved in the biosynthesis of heme, is inhibited by Pb [15, 16, 27]. In the present exome-wide screening analysis, we tested two SNPs (rs1805313 and rs1800435) of *ALAD* and 5 SNPs (rs2642926, rs3755652, rs4973768, rs75615379, and rs9854207) of *SLC4A7*; however, these SNPs did not achieve the suggestive level of association applied in this study (*P* < 1 × 10^−4^). This discrepancy may be attributed to the ethnic differences in terms of heavy metal levels [28] and genetic polymorphisms of heavy-metal-related genes [29]. A meta-analysis suggested that a significant association between *ALAD* genetic polymorphisms and blood Pb level is limited only to the high-Pb exposure group [27]. These results suggest that the role of genetic factors in determining the body burden of Pb may vary according to the level of environmental exposure.

In the present study, we demonstrated a suggestively significant association between *C12orf51* rs11066280, *MYL2* rs12229654, and *ALDH2* rs671 polymorphisms and blood Pb levels in 1,481 Korean subjects. Interestingly, all three SNPs were highly related to alcohol consumption [21, 24]. *ALDH2* rs671, a non-synonymous SNP that occurs within a coding region, is located at position 487 (Glu > Lys), while *C12orf51* rs11066280 and *MYL2* rs12229654 are located in the intron and intergenic regions, respectively. Individuals with the *ALDH2* rs671 variant allele exhibit markedly decreased ALDH2 enzyme activity and inhibited detoxification of toxic acetaldehyde, which is the first and most toxic metabolite of ethanol [24, 30]. Therefore, alcohol drinking behavior and dependency are associated with the *ALDH2* rs671 SNP [31, 32]. Although *C12orf51* rs11066280 and *MYL2* rs12229654 were not located in a functional element, these two SNPs exhibit strong linkage disequilibrium with SNPs associated with alcohol drinking behavior [21, 33]. In our data, no differences in dietary Pb intake according to the genotypes of these alcohol-related genes were found; however, a difference was observed in the correlation of dietary Pb intake and blood Pb level according to these genotypes. Moreover, when stratified by drinking status, a potential association of *C12orf51* rs11066280, *MYL2* rs12229654, and *ALDH2* rs671 with blood Pb level was observed only in drinkers. A marginally significant gene-environment interaction between *ALDH2* rs671 and alcohol drinking on blood Pb level was found. Therefore, our data indicate that the association between the three SNPs investigated and blood Pb levels is mediated by alcohol drinking behavior.

Numerous studies have established links between higher blood Pb levels and alcohol consumption [13, 23, 34–36]. Consistent with their findings, the present data also found increased levels of blood Pb in alcohol drinkers (2.26 μg/dL) relative to non-drinkers (2.07 μg/dL). In addition, dietary Pb exposure level of drinkers (20.77 μg/day) was significantly higher than in non-drinkers (19.36 μg/day), although alcoholic beverages have low Pb concentrations (11.13 μg/kg) and contribute to only about 6.5% of total dietary Pb intake. These findings indicate that an increase in blood Pb levels in alcohol drinkers, rather than an increase in direct exposure to Pb via alcohol consumption, is associated with a variety of unhealthy lifestyle behaviors as a result of clustering tendency [37, 38]. Various unhealthy behaviors are considered to act as pivotal factors in increasing blood Pb concentrations.

In addition, it is considered that several possible biological mechanisms, such as the regulation of iron metabolism [39], decreased immunity [40, 41], and increased calcium excretion [23], underlie the effects of alcohol drinking on increased blood Pb levels. Firstly, alcohol consumption greatly increases Pb absorption by damaging the body’s ability to regulate the absorption of iron. Further, the levels of hepcidin, which is involved in iron metabolism, are decreased by alcohol consumption. As iron and Pb compete for absorption, alcohol consumption may result in increased Pb absorption [39]. In addition, Pb is eliminated by macrophages, and it has recently been reported that macrophages and monocytes remove nanoparticles of specific heavy metals [42, 43]. Although it is well known that chronic Pb exposure decreases immune function [40, 41], our data suggest that a decrease in immunity, caused by a predisposing factor such as high alcohol intake, may increase the absorption of Pb. Lastly, calcium is one of the most important nutritional factors in relation to the uptake of heavy metals, and calcium homeostasis is disrupted by alcohol consumption [44]; calcium deficiency resulting from alcohol consumption increases Pb absorption as the two ions are transported completely by the same transporter [45].

In this study, the effect of three alcohol-related SNPs on blood Pb levels was found to be dependent on the daily levels of calcium intake. Significant genetic effects on blood Pb levels were observed in the low calcium-intake group but not in the high calcium-intake group. Our data were consistent with the findings of Pizent et al., who reported that alcohol consumption and low calcium intake are associated with increased blood Pb levels [23]. These data indicate that genetic susceptibility to high blood Pb levels may be mitigated if calcium depletion due to alcohol consumption were appropriately compensated by the intake of calcium-rich foods or supplements.

This study has strengths and limitations; the main strength was the use of a representative sample of the general Korean population with no history of occupational exposure to Pb. Moreover, this study used the probable dietary intake levels of Pb to test the independent genetic effect. This study has several limitations, such as the limited statistical power of exome-wide screening because of the small sample size. To overcome this limitation, we used a two-stage approach, involving screening and replication; this increased power for the detection of genetic associations. In addition, the Bonferroni correction was applied for multiple tests, and a threshold for statistical significance was set. Nonetheless, none of SNPs showed an association with exome-wide significance (*P* < 1 × 10^−6^) in the screening stage. Four SNPs showed a potential association with blood Pb levels and achieved the level of suggestive association used in this study. Therefore, we could not exclude the possibility of false positives in this process. However, the same associations were confirmed in the 2-step test as well. Moreover, the biological plausibility of association between the suggested SNPs and the variation of blood Pb concentration in humans supports our results. Another limitation of this study is that we did not conduct the replication analysis in another independent cohort; therefore, the SNPs identified in the current study require replication in independent cohorts.

## Conclusions

The present exome-wide association study demonstrates that *C12orf51* rs11066280, *MYL2* rs12229654, and *ALDH2* rs671 polymorphisms are linked to blood Pb levels in the Korean population. These three SNPs are involved in the determination of the Pb body burden in Koreans via regulation of alcohol drinking behavior, and their negative effects may be compensated by appropriate calcium intake. Our results suggest that individuals with genetic susceptibility to elevated Pb body burden must take measures to avoid environmental exposure to Pb and maintain a healthy lifestyle, that is, avoid smoking and drinking and appropriate calcium intake.

## Additional file

Additional file 1: Results of exome-wide association study for screening of genetic variability for blood lead levels; **Table S1**. Top 100 single nucleotide polymorphisms most significantly associated with blood lead levels, as identified by the exome-wide association study; **Figure S1**. Regional association plot of single nucleotide polymorphisms near *C12orf51* and *ALDH2* genes on chromosome 12q24. (DOCX 139 kb)

## Abbreviations

ALAD: Aminolevulinate dehydratase
ALDH2: Aldehyde dehydrogenase 2
C12orf51: Chromosome 12 open reading frame 51
GM: Geometric mean
GSD: Geometric standard deviation
GWAS: Genome-wide association study
HWE: Hardy-Weinberg equilibrium
MAF: Minor allele frequency
MYL2: Myosin light chain 2
SLC4A7: Solute carrier family 4, sodium bicarbonate cotransporter, member 7
SNP: Single nucleotide polymorphism
VDR: Vitamin D receptor
