# Supplementary material for: Exome-wide association study identifies genetic polymorphisms of C12orf51, MYL2, and ALDH2 associated with blood lead levels in the general Korean population
Source: Environ Health. 2017 Feb 17;16:11. doi: 10.1186/s12940-017-0220-x (PMC5316181; doi:10.1186/s12940-017-0220-x)
Supplement: Additional file 1: — Results of exome-wide association study for screening of genetic variability for blood lead levels; Table S1. Top 100 single nucleotide polymorphisms most significantly associated with blood lead levels, as identified by the exome-wide association study; Figure S1. Regional association plot of single nucleotide polymorphisms near C12orf51 and ALDH2 genes on chromosome 12q24. (DOCX 139 kb) [file 12940_2017_220_MOESM1_ESM.docx]

**Table S1.** Top 100 single nucleotide polymorphisms most significantly associated with blood lead level, as identified by the exome-wide association study

| **rs ID** | **CHR** | **BP** | **Gene symbol** | **Functional location** | **Amino acid change** | **Major allele** | **Minor allele** | **MAF** | **P** | **BETA** | **SE** | **P** |
| --- | --- | --- | --- | --- | --- | --- | --- | --- | --- | --- | --- | --- |
| rs11066280 | 12 | 111302166 | *C12orf51* | intronic | . | T | A | 0.170 | 0.752 | -0.331 | 0.070 | 2.88.E-06 |
| rs2074356 | 12 | 111129784 | *C12orf51* | intronic | . | G | A | 0.140 | 0.357 | -0.348 | 0.074 | 2.98.E-06 |
| rs11066015 | 12 | 110652392 | *ACAD10* | intronic | . | G | A | 0.156 | 0.497 | -0.329 | 0.071 | 3.97.E-06 |
| rs671 | 12 | 110726149 | *ALDH2* | exonic | nonsynonymous_SNV | G | A | 0.155 | 0.493 | -0.328 | 0.071 | 4.40.E-06 |
| rs3782886 | 12 | 110594872 | *BRAP* | exonic | synonymous_SNV | A | G | 0.165 | 0.871 | -0.319 | 0.070 | 6.27.E-06 |
| rs12229654 | 12 | 109898844 | *MYL2(dist=39211),CUX2(dist=57367)* | intergenic | . | A | C | 0.139 | 0.852 | -0.331 | 0.075 | 1.28.E-05 |
| rs2228539 | 19 | 6877378 | *EMR1;EMR1* | exonic;splicing | nonsynonymous_SNV | A | G | 0.015 | 1.000 | 0.903 | 0.215 | 3.18.E-05 |
| rs2745099 | 16 | 1477459 | *PTX4* | exonic | nonsynonymous_SNV | C | A | 0.095 | 0.186 | -0.354 | 0.086 | 4.80.E-05 |
| rs41268474 | 1 | 150959136 | *C1orf68* | exonic | nonsynonymous_SNV | G | A | 0.058 | 0.679 | 0.441 | 0.111 | 7.90.E-05 |
| rs2667672 | 16 | 1476381 | *PTX4* | exonic | nonsynonymous_SNV | C | A | 0.118 | 0.664 | -0.317 | 0.080 | 8.76.E-05 |
| rs2745097 | 16 | 1476500 | *PTX4* | exonic | nonsynonymous_SNV | C | A | 0.118 | 0.664 | -0.317 | 0.080 | 8.76.E-05 |
| rs6126559 | 20 | 35990690 | *VSTM2L* | intronic | . | G | A | 0.401 | 0.161 | 0.217 | 0.055 | 9.50.E-05 |
| rs151124111 | 14 | 24029155 | *AP1G2* | exonic | nonsynonymous_SNV | G | A | 0.010 | 0.045 | 0.918 | 0.240 | 1.44.E-04 |
| rs9658588 | 1 | 85821247 | *CYR61* | exonic | synonymous_SNV | G | A | 0.022 | 0.212 | 0.651 | 0.172 | 1.68.E-04 |
| rs3803185 | 13 | 49103026 | *ARL11* | exonic | nonsynonymous_SNV | A | G | 0.177 | 0.538 | 0.264 | 0.070 | 1.76.E-04 |
| rs2234028 | 6 | 42821252 | *TBCC* | exonic | nonsynonymous_SNV | G | A | 0.065 | 0.711 | 0.406 | 0.108 | 1.97.E-04 |
| rs2234027 | 6 | 42821285 | *TBCC* | exonic | nonsynonymous_SNV | G | A | 0.065 | 0.711 | 0.406 | 0.108 | 1.97.E-04 |
| rs138147609 | 16 | 28603710 | *SULT1A2* | exonic | stopgain_SNV | C | A | 0.018 | 1.000 | 0.741 | 0.198 | 2.05.E-04 |
| rs12793348 | 11 | 93552684 | *PANX1* | exonic | nonsynonymous_SNV | A | G | 0.243 | 0.392 | 0.221 | 0.060 | 2.48.E-04 |
| rs2290558 | 15 | 39955626 | *SPTBN5* | exonic | nonsynonymous_SNV | C | G | 0.035 | 0.463 | 0.517 | 0.140 | 2.55.E-04 |
| rs2248020 | 11 | 92899613 | *C11orf75* | intronic | . | C | A | 0.385 | 0.704 | -0.198 | 0.054 | 2.91.E-04 |
| rs4057749 | 11 | 123799446 | *OR8B4* | exonic | nonsynonymous_SNV | A | G | 0.190 | 0.558 | 0.235 | 0.066 | 4.00.E-04 |
| rs634501 | 5 | 180151274 | *MGAT1* | exonic | nonsynonymous_SNV | G | A | 0.390 | 0.072 | 0.184 | 0.052 | 4.17.E-04 |
| rs118034043 | 9 | 138839708 | *UBAC1* | exonic | nonsynonymous_SNV | G | A | 0.011 | 1.000 | 0.892 | 0.252 | 4.30.E-04 |
| rs7998781 | 13 | 48036911 | *RCBTB2(dist=31594),CYSLTR2(dist=142041)* | intergenic | . | G | A | 0.160 | 0.246 | 0.258 | 0.073 | 4.85.E-04 |
| rs2230301 | 1 | 218264248 | *EPRS* | exonic | nonsynonymous_SNV | A | C | 0.086 | 1.000 | -0.331 | 0.095 | 5.11.E-04 |
| rs75766177 | 19 | 36674533 | *ZNF565* | exonic | nonsynonymous_SNV | C | A | 0.091 | 1.000 | 0.318 | 0.091 | 5.42.E-04 |
| rs12365708 | 11 | 68269119 | *MTL5* | exonic | nonsynonymous_SNV | A | G | 0.044 | 0.615 | 0.453 | 0.132 | 6.30.E-04 |
| rs148031091 | 4 | 158091771 | *GLRB* | exonic | nonsynonymous_SNV | G | C | 0.010 | 1.000 | 0.912 | 0.265 | 6.32.E-04 |
| rs7016250 | 8 | 65655983 | *BHLHE22* | exonic | nonsynonymous_SNV | C | A | 0.353 | 0.492 | 0.192 | 0.056 | 6.34.E-04 |
| rs712270 | 17 | 17997892 | *MYO15A* | exonic | nonsynonymous_SNV | A | T | 0.122 | 0.833 | 0.274 | 0.080 | 6.42.E-04 |
| rs139478067 | 7 | 92760823 | *SAMD9L* | exonic | nonsynonymous_SNV | G | A | 0.011 | 0.055 | 0.787 | 0.231 | 7.07.E-04 |
| rs925368 | 12 | 108875362 | *GIT2* | exonic | nonsynonymous_SNV | A | G | 0.061 | 1.000 | -0.376 | 0.111 | 7.46.E-04 |
| rs7807131 | 7 | 94839068 | *PON3* | intronic | . | G | A | 0.038 | 0.521 | 0.461 | 0.136 | 7.62.E-04 |
| rs117152313 | 9 | 124544697 | *DAB2IP* | exonic | nonsynonymous_SNV | G | A | 0.052 | 0.637 | 0.393 | 0.116 | 7.86.E-04 |
| rs139627756 | 2 | 55543087 | *CCDC88A* | exonic | nonsynonymous_SNV | A | G | 0.011 | 1.000 | 0.849 | 0.252 | 8.07.E-04 |
| rs10278730 | 7 | 138211800 | *KIAA1549* | intronic | . | A | G | 0.340 | 0.842 | -0.188 | 0.056 | 8.07.E-04 |
| rs13024907 | 2 | 46139028 | *PRKCE* | intronic | . | G | A | 0.412 | 0.853 | 0.180 | 0.054 | 8.61.E-04 |
| rs1000203 | 14 | 39965858 | *FBXO33(dist=994404),LRFN5(dist=1180656)* | intergenic | . | A | G | 0.150 | 0.295 | 0.241 | 0.072 | 8.67.E-04 |
| rs2233213 | 5 | 52433762 | *MOCS2* | exonic | nonsynonymous_SNV | A | G | 0.091 | 1.000 | 0.305 | 0.091 | 8.90.E-04 |
| rs17744093 | 2 | 29148258 | *C2orf71* | exonic | nonsynonymous_SNV | G | C | 0.138 | 1.000 | 0.255 | 0.076 | 9.01.E-04 |
| rs12411176 | 1 | 110567989 | *KCNC4* | exonic | nonsynonymous_SNV | G | A | 0.117 | 0.830 | -0.273 | 0.082 | 9.04.E-04 |
| rs2865531 | 16 | 73947817 | *CFDP1* | intronic | . | T | A | 0.490 | 0.523 | -0.183 | 0.055 | 9.05.E-04 |
| rs4621553 | 5 | 113058063 | *YTHDC2(dist=99180),KCNN2(dist=667852)* | intergenic | . | A | G | 0.057 | 0.213 | -0.368 | 0.111 | 9.82.E-04 |
| rs6457617 | 6 | 32771829 | *HLA-DQB1(dist=29385),HLA-DQA2(dist=45312)* | intergenic | . | A | G | 0.430 | 0.067 | 0.168 | 0.051 | 1.02.E-03 |
| rs6457620 | 6 | 32771977 | *HLA-DQB1(dist=29533),HLA-DQA2(dist=45164)* | intergenic | . | C | G | 0.430 | 0.067 | 0.168 | 0.051 | 1.02.E-03 |
| rs73003074 | 3 | 150391810 | *FAM194A* | exonic | nonsynonymous_SNV | A | C | 0.088 | 0.252 | 0.298 | 0.090 | 1.03.E-03 |
| rs2303975 | 11 | 14233575 | *SPON1* | intronic | . | G | A | 0.099 | 0.804 | -0.289 | 0.088 | 1.08.E-03 |
| rs2303690 | 19 | 53217319 | *ELSPBP1* | exonic | nonsynonymous_SNV | A | G | 0.120 | 0.397 | -0.261 | 0.079 | 1.08.E-03 |
| rs2220948 | 1 | 96883528 | *FLJ31662(dist=1166028),PTBP2(dist=76235)* | intergenic | . | G | A | 0.421 | 0.142 | 0.169 | 0.052 | 1.10.E-03 |
| rs3731625 | 2 | 24292552 | *ITSN2* | exonic | nonsynonymous_SNV | A | G | 0.095 | 1.000 | -0.294 | 0.090 | 1.16.E-03 |
| rs2231682 | 10 | 101473789 | *COX15* | exonic | nonsynonymous_SNV | G | A | 0.013 | 1.000 | 0.762 | 0.233 | 1.16.E-03 |
| rs34141181 | 1 | 40478014 | *RLF* | exonic | nonsynonymous_SNV | C | G | 0.026 | 1.000 | -0.542 | 0.166 | 1.20.E-03 |
| rs2228145 | 1 | 152693594 | *IL6R* | exonic | nonsynonymous_SNV | A | C | 0.433 | 0.784 | -0.174 | 0.053 | 1.20.E-03 |
| rs9294631 | 6 | 65679184 | *EYS* | exonic | nonsynonymous_SNV | A | G | 0.430 | 0.784 | -0.174 | 0.054 | 1.23.E-03 |
| rs1079166 | 19 | 18246214 | *KIAA1683* | splicing | . | A | G | 0.395 | 0.851 | -0.176 | 0.054 | 1.23.E-03 |
| rs2568076 | 11 | 8905191 | *C11orf16* | exonic | nonsynonymous_SNV | G | A | 0.122 | 0.032 | -0.248 | 0.076 | 1.23.E-03 |
| rs11061330 | 12 | 130160516 | *GPR133* | intronic | . | A | G | 0.335 | 0.615 | 0.178 | 0.055 | 1.29.E-03 |
| rs7747960 | 6 | 152833167 | *SYNE1* | intronic | . | C | A | 0.385 | 0.777 | 0.172 | 0.054 | 1.41.E-03 |
| rs2070955 | 1 | 24067360 | *FUCA1* | exonic | nonsynonymous_SNV | G | A | 0.205 | 1.000 | 0.209 | 0.065 | 1.41.E-03 |
| rs2229579 | 1 | 24073749 | *CNR2* | exonic | nonsynonymous_SNV | G | A | 0.205 | 1.000 | 0.209 | 0.065 | 1.41.E-03 |
| rs183909467 | 14 | 93290938 | *GOLGA5* | exonic | nonsynonymous_SNV | G | C | 0.014 | 1.000 | -0.721 | 0.225 | 1.42.E-03 |
| rs6699417 | 1 | 88896031 | *LOC100505768(dist=1286105),PKN2(dist=26479)* | intergenic | . | A | G | 0.457 | 0.175 | -0.165 | 0.052 | 1.42.E-03 |
| rs2436493 | 19 | 5728323 | *TMEM146* | intronic | . | A | G | 0.456 | 0.928 | 0.170 | 0.053 | 1.44.E-03 |
| rs12110273 | 5 | 4341545 | *IRX1(dist=687028),LOC340094(dist=745927)* | intergenic | . | A | G | 0.344 | 0.766 | 0.176 | 0.055 | 1.45.E-03 |
| rs883079 | 12 | 113277623 | *TBX5* | UTR3 | . | G | A | 0.429 | 1.000 | -0.170 | 0.053 | 1.47.E-03 |
| rs885389 | 12 | 130187715 | *GPR133* | intronic | . | A | G | 0.427 | 0.582 | 0.175 | 0.055 | 1.47.E-03 |
| rs117674897 | 2 | 210887734 | *C2orf67* | exonic | nonsynonymous_SNV | A | G | 0.021 | 1.000 | -0.588 | 0.185 | 1.59.E-03 |
| rs9453108 | 6 | 65677579 | *EYS* | intronic | . | A | G | 0.432 | 0.715 | -0.170 | 0.054 | 1.60.E-03 |
| rs12681691 | 8 | 134587315 | *ST3GAL1* | intronic | . | A | G | 0.318 | 0.679 | -0.178 | 0.056 | 1.64.E-03 |
| rs4129267 | 1 | 152692888 | *IL6R* | intronic | . | G | A | 0.432 | 0.855 | -0.169 | 0.053 | 1.64.E-03 |
| rs11058388 | 12 | 124966104 | *TMEM132B(dist=256562),LOC400084(dist=43083)* | intergenic | . | A | G | 0.462 | 0.588 | 0.166 | 0.052 | 1.66.E-03 |
| rs1864183 | 5 | 81584972 | *ATG10* | exonic | nonsynonymous_SNV | A | G | 0.081 | 1.000 | 0.305 | 0.097 | 1.67.E-03 |
| rs2273664 | 2 | 32836984 | *TTC27* | exonic | nonsynonymous_SNV | G | A | 0.235 | 0.382 | 0.192 | 0.061 | 1.69.E-03 |
| rs4734653 | 8 | 103732990 | *KLF10* | exonic | nonsynonymous_SNV | G | A | 0.126 | 1.000 | -0.248 | 0.079 | 1.79.E-03 |
| rs12028397 | 1 | 3937525 | *LOC728716(dist=25022),LOC284661(dist=434446)* | intergenic | . | G | A | 0.449 | 0.928 | -0.167 | 0.053 | 1.86.E-03 |
| rs2576696 | 2 | 55359185 | *MTIF2(dist=9297),PRORSD1P(dist=3774)* | intergenic | . | C | A | 0.298 | 0.748 | 0.181 | 0.058 | 1.89.E-03 |
| rs946185 | 10 | 76093745 | *ADK* | intronic | . | G | A | 0.328 | 0.052 | -0.167 | 0.054 | 1.90.E-03 |
| rs4537545 | 1 | 152685503 | *IL6R* | intronic | . | G | A | 0.436 | 0.648 | -0.167 | 0.054 | 1.91.E-03 |
| rs10938397 | 4 | 44877284 | *GNPDA2(dist=453915),GABRG1(dist=855260)* | intergenic | . | A | G | 0.274 | 0.310 | -0.180 | 0.058 | 1.92.E-03 |
| rs1801197 | 7 | 92893689 | *CALCR* | exonic | nonsynonymous_SNV | G | A | 0.114 | 0.502 | 0.254 | 0.082 | 1.93.E-03 |
| rs12641981 | 4 | 44874640 | *GNPDA2(dist=451271),GABRG1(dist=857904)* | intergenic | . | G | A | 0.273 | 0.259 | -0.179 | 0.058 | 1.95.E-03 |
| rs10485165 | 6 | 89169536 | *CNR1(dist=237050),RNGTT(dist=207172)* | intergenic | . | G | A | 0.293 | 0.590 | 0.182 | 0.058 | 2.00.E-03 |
| rs139208500 | 19 | 9049991 | *MUC16* | exonic | nonsynonymous_SNV | G | A | 0.012 | 1.000 | 0.751 | 0.242 | 2.01.E-03 |
| rs17150488 | 5 | 101802354 | *SLCO6A1* | exonic | nonsynonymous_SNV | A | G | 0.015 | 1.000 | 0.673 | 0.217 | 2.02.E-03 |
| rs77695791 | 12 | 120601014 | *GCN1L1* | exonic | nonsynonymous_SNV | G | A | 0.029 | 1.000 | 0.490 | 0.158 | 2.09.E-03 |
| rs3814848 | 14 | 89720786 | *KCNK13* | exonic | nonsynonymous_SNV | G | A | 0.087 | 0.400 | -0.285 | 0.092 | 2.09.E-03 |
| rs117238101 | 19 | 47844955 | *GPR77* | exonic | nonsynonymous_SNV | G | A | 0.020 | 1.000 | 0.585 | 0.190 | 2.14.E-03 |
| rs59506448 | 5 | 79067488 | *CMYA5* | exonic | nonsynonymous_SNV | C | A | 0.011 | 1.000 | 0.778 | 0.252 | 2.16.E-03 |
| rs6790837 | 3 | 126215308 | *HEG1* | exonic | nonsynonymous_SNV | A | G | 0.478 | 0.151 | 0.169 | 0.055 | 2.17.E-03 |
| rs2657291 | 10 | 76596709 | *SAMD8* | intronic | . | G | A | 0.083 | 0.764 | -0.291 | 0.095 | 2.18.E-03 |
| rs78280171 | 4 | 175898555 | *ADAM29* | exonic | nonsynonymous_SNV | G | A | 0.233 | 0.209 | 0.188 | 0.061 | 2.19.E-03 |
| rs56272205 | 7 | 127786881 | *PRRT4* | exonic | nonsynonymous_SNV | G | A | 0.133 | 0.434 | 0.233 | 0.076 | 2.26.E-03 |
| rs61730330 | 6 | 29749308 | *ZFP57* | exonic | nonsynonymous_SNV | G | A | 0.010 | 1.000 | 0.810 | 0.265 | 2.34.E-03 |
| rs9551080 | 13 | 23675773 | *SPATA13* | intronic | . | G | A | 0.307 | 0.833 | 0.176 | 0.057 | 2.36.E-03 |
| rs56279116 | 5 | 132038071 | *IL4* | exonic | nonsynonymous_SNV | G | A | 0.015 | 1.000 | 0.664 | 0.217 | 2.37.E-03 |
| rs2483350 | 1 | 112775306 | *CTTNBP2NL* | intronic | . | A | C | 0.499 | 0.281 | 0.165 | 0.054 | 2.38.E-03 |
| rs13225097 | 7 | 149938100 | *GIMAP4(dist=36126),GIMAP6(dist=15297)* | intergenic | . | A | G | 0.197 | 0.777 | 0.201 | 0.066 | 2.40.E-03 |
| rs62038760 | 16 | 11795130 | *ZC3H7A* | intronic | . | A | G | 0.452 | 0.650 | -0.159 | 0.052 | 2.47.E-03 |
| rs35562811 | 20 | 34005818 | *SCAND1* | exonic | nonsynonymous_SNV | C | G | 0.024 | 0.029 | 0.489 | 0.161 | 2.48.E-03 |

**
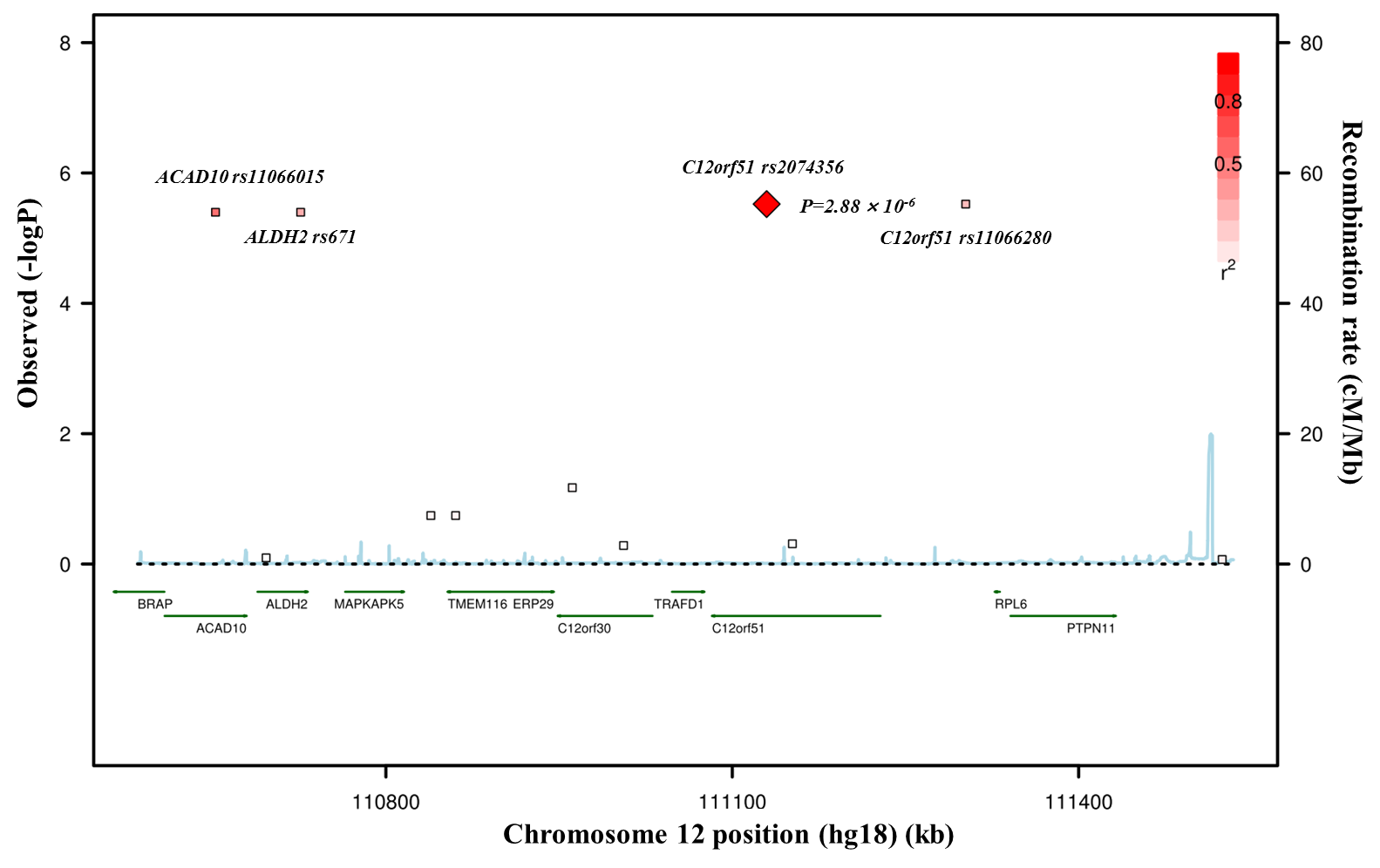
**

**Figure S1.** Regional association plot of single nucleotide polymorphisms near C12orf51 and ALDH2 genes on chromosome 12q24
